# The HAND Database: a gateway to understanding the role of HIV in HIV-associated neurocognitive disorders

**DOI:** 10.1186/s12920-015-0143-8

**Published:** 2015-10-28

**Authors:** Tess Z Griffin, Weiliang Kang, Yongjie Ma, Ming Zhang

**Affiliations:** Department of Epidemiology and Biostatistics, University of Georgia, Athens, GA 30602 USA; Institute of Bioinformatics, University of Georgia, Athens, GA 30602 USA; Medical University Cancer Institute and Hospital, National Clinical Research Center of Cancer, Key laboratory of Cancer Prevention and Therapy, Tianjin, 300060 PR China; Present address: College of Pharmacy, University of Illinois, Chicago, IL 60612 USA

**Keywords:** HIV, HIV-associated neurocognitive disorder, Dementia, Genotyping, Quality control

## Abstract

**Background:**

Despite an augmented research effort and scale-up of highly active antiretroviral therapy, a high prevalence of HIV-1-associated neurocognitive disorders (HAND) persists in the HIV-infected population. Nearly 50 % of all HIV-1-infected individuals suffer from a neurocognitive disorder due to neural and synaptodendritic damage. Challenges in HAND research, including limited availability of brain tissue from HIV patients, variation in HAND study protocols, and virus genotyping inconsistency and errors, however, have resulted in studies with insufficient power to delineate molecular mechanisms underlying HAND pathogenesis. There exists, therefore, a great need for a reliable and centralized resource specific to HAND research, particularly for epidemiological study and surveillance in resource-limited countries where severe forms of HAND persist.

**Description:**

To address the aforementioned imperative need, here we present the HAND Database, a resource containing well-curated and up-to-date HAND virus information and associated clinical and epidemiological data. This database provides information on 5,783 non-redundant HIV-1 sequences from global HAND research published to date, representing a total of 163 unique individuals that have been assessed for HAND. A user-friendly interface allows for flexible searching, filtering, browsing, and downloading of data. The most comprehensive database of its kind, the HAND Database not only bolsters current HAND research by increasing sampling power and reducing study biases caused by protocol variation and genotyping inconsistency, it allows for comparison between HAND studies across different dimensions. Development of the HAND Database has also revealed significant knowledge gaps in HIV-driven neuropathology. These gaps include inadequate sequencing of viral genes beyond *env*, lack of HAND viral data from HIV epidemiologically important regions including Asian and Sub-Saharan African countries, and biased sampling toward the male gender, all factors that impede efforts toward providing an improved quality of life to HIV-infected individuals, and toward elimination of viruses in the brain.

**Conclusion:**

Our aim with the HAND database is to provide researchers in both the HIV and neuroscience fields a comprehensive and rigorous data source toward better understanding virus compartmentalization and to help in design of improved strategies against HAND viruses. We also expect this resource, which will be updated on a regular basis, to be useful as a reliable reference for further HAND epidemiology studies. The HAND Database is freely available and accessible online at http://www.handdatabase.org.

## Background

Human immunodeficiency virus (HIV)-associated neurocognitive disorder (HAND) occurs due to damage to neurons and synapses by viral protein products, and due to a chemokine/cytokine imbalance in the brain, a pro-inflammatory response to HIV infection of macrophages and microglia [1–3]. HIV entry into the brain is an early event following infection [4], and presence of the blood brain barrier greatly limits entry of antiretroviral therapy into the brain. Our ability to control viral levels within and viral damage to the HIV-infected brain, therefore, remains highly limited. While the introduction of highly active antiretroviral therapy (HAART) brought about a decrease in the incidence of the most severe forms of HAND, i.e., HIV-associated dementia, the prevalence of milder forms has continued to increase [5–7]. In the recent HIV Anti-Retroviral Therapy Effects Research Study, nearly 50 % of all HIV-1 individuals exhibited some form of HAND, including deficits in motor function, verbal fluency, learning, memory, and attention [8]. HAND individuals experience difficulty performing day-to-day tasks, are less likely to adhere to medical treatments and other HIV-1 prevention practices, and ultimately suffer from around a threefold increased risk of death as compared to a mentally-healthy HIV-1 individual [9]. In addition, in resource-limited countries, the most severe forms of HAND continue to devastate the mental health of HIV individuals [9].

Delineating the underpinning molecular mechanisms of HAND development is critical to providing HIV-infected individuals an elevated quality of life, as well as toward clearance of the virus repertoire in the brain. Research in this area, however, has been largely limited by availability of samples from both the brain and from HAND-assessed individuals. In addition, a need to understand HAND progression across an HIV individual’s lifespan, coupled with difficulty in obtaining brain samples, has made cerebrospinal fluid (CSF) sampling a surrogate endpoint for assessing HAND development [10]. Both, small sample size from individual studies and indirect CSF inference have made it difficult to fully assess the complex interaction between viruses and the brain in the HAND setting. Additionally, variations in study methodologies and result interpretations have further confounded HAND studies, leading to conflicting findings in the field. To address these issues, there therefore exists a great need for a reliable HIV sequence resource, of adequate sample size, for HAND research.

Toward this effort, we developed a centralized HAND Database based on all HAND studies published to date. This resource database is freely accessible at: http://www.handdatabase.org. The HAND Database serves as the most comprehensive database in its field, and contains well-curated HAND virus information, epidemiology sampling data, patient clinical status, and therapy treatment information. All information was cross-validated using multiple resources, including the literature, GenBank entry, and author contact. Furthermore, all viral sequences have undergone stringent quality control examination, including genotyping validation, in order to minimize genotyping errors frequently seen in HIV subtype-based studies [11].

The only other published HIV database related to brain tissue, The HIV Brain Sequence Database [12], contains HIV *env* sequences from brain tissue, as well as from other tissues in patients with brain samples. In contrast, our database contains HAND-specific information with regards to virus sequences (genome coverage beyond *env*), epidemiology sampling information, clinical data, and treatment status, all factors important to the study of HAND pathogenesis. Unprecedented in its comprehensiveness of curated HAND HIV information, our HAND Database serves as a centralized gateway to study the role of HIV in the HAND setting.

## Construction and content

### Data sources

An extensive literature review was conducted to develop a comprehensive set of HAND-related research articles, from which we then extracted sequence data from HAND-assessed individuals. This literature search resulted in the use of data from 41 published studies. Publically available HIV-1 sequence data were collected from the GenBank (last accessed 3/2013) and the LANL HIV sequence database (last accessed 2/2014) [13, 14]. HIV-1 individual sampling and clinical information was collected from the relevant literature, the two aforementioned databases, and through communication with publication authors.

### Sequence and clinical data filtering

All collected sequence data were validated through a series of quality control steps. We first employed the LANL quality control pipeline to check for potential problematic viruses with sequencing errors [13]. Amplification contamination was detected using BLASTn (v. 2.2.26) [15]. In addition, data regarding epidemiology sampling, clinical status, and treatment status were cross-referenced whenever available in more than one of the resources listed above.

### Genotyping analysis

Genotyping of HIV sequence data is frequently inconsistent and error-prone [11].

Therefore, all filtered HIV sequences were re-genotyped. Here we applied the jumping profile Hidden Markov Model genotyping program (jpHMM), whose genotyping accuracy has been established [16–18]. In brief, following a hypermutation analysis [13], sequences greater than 300 nucleotides in length and with a hypermutation p-value of 0.05 or greater were subject to genotyping.

### Database schema

The HAND Database was constructed using the relational database management system MySQL (v.5.6.17). MySQL was chosen for its ease of use, its high reliability, and as it is freely available. HIV-1 sequence and clinical data were compiled into one flat file, with annotations divided into three major categories: sequence and sequence descriptor data, HIV-1 patient descriptor data, and sample descriptor data (Table 1). Sequence data included the HIV-1 nucleotide sequence, sequence accession number, sequence genotype information, and sequence length. Epidemiology data included the geographical location and year at time of sampling, as well as tissue sampled. Patient data at time of sampling included patient age, risk factor, health status, CD4 count, viral load, HIV treatment information (treatment status, and when applicable, treatment type and duration), and patient HAND information (HAND status, the presence or absence of HAND, and when applicable, HAND type).

**Table 1:** Overview of database annotations

Category	Annotation	Example	Explanation
Patient	Patient: Code	SUBJECT_4	The originating publication ID for this patient was “SUBJECT_4”
Patient	Patient: HAND Status	HIVE + ADC	This patient had two forms of HAND: HIVE and ADC
Patient	Patient: Sex	M	This patient was male
Patient	Patient: Risk Factor	IV Drug User	This patient may have contracted the HIV virus through IV drug use
Patient	Patient: Viral Load At Sampling	Unknown Viral Load	Information on this patient’s viral load at time of sampling could not be found
Patient	Patient: CD4 Count At Sampling	5 cells/ul	This patient had a CD4 count of 5 cells/ul at time of sampling
Patient	Patient: HIV Therapy Status	AZT	This patient had received AZT therapy prior to sampling
Patient	Patient: HIV Therapy Months	20	This patient had received HIV therapy for 20 months prior to sampling
Patient	Patient: Age At Sampling	29	This patient was 29 years of age at time of sampling
Patient	Patient: Health At Sampling	AIDS	This patient had AIDS at time of sampling
Sampling	Sampling: Year	1993	This sample was obtained in the year 1993
Sampling	Sampling: Country (City)	Japan (unknown)	This sample was obtained in an unknown city in the country of Japan
Sampling	Sampling: Tissue	Spleen	This sample was obtained from spleen tissue
Sequence	Sequence: Polyprotein (Protein)	Tat (gp160)	This sequence segment covers the Tat and Gp160 HIV regions
Sequence	Sequence: Genotyping Information^1^	B (B)*	This sequence was listed by the originating data source as a B subtype sequence. jpHMM genotyping confirmed subtype as B. Recombination testing detected recombination events in this sequence (indicated by the asterisk).
Sequence	Sequence: Accession Number	U82096	The sequence accession number for this sequence is U82096
Sequence	Sequence: PMID	9718130	The sequence PMID for this sequence is 9718130
Sequence	Sequence: Length in Nucleotides	150	The sequence length for this sequence segment is 150 nucleotides
Sequence	HIV Sequence	cccaaat…	The nucleotide sequence for this sequence is “cccaaat…”

^1^The sequence genotyping annotation provides genotyping and recombination information in the following format: Subtype as reported in the original source material (Subtype as reported by jpHMM genotyping) and recombination information. Additional symbols used in this annotation include: “#” = not tested due to insufficient length of sequence, “*” = *p* < 0.05 for recombination test, “(No)” = *p* > 0.05 for recombination test

## Utility

### Database access and web query interface

**Fig. 1:**
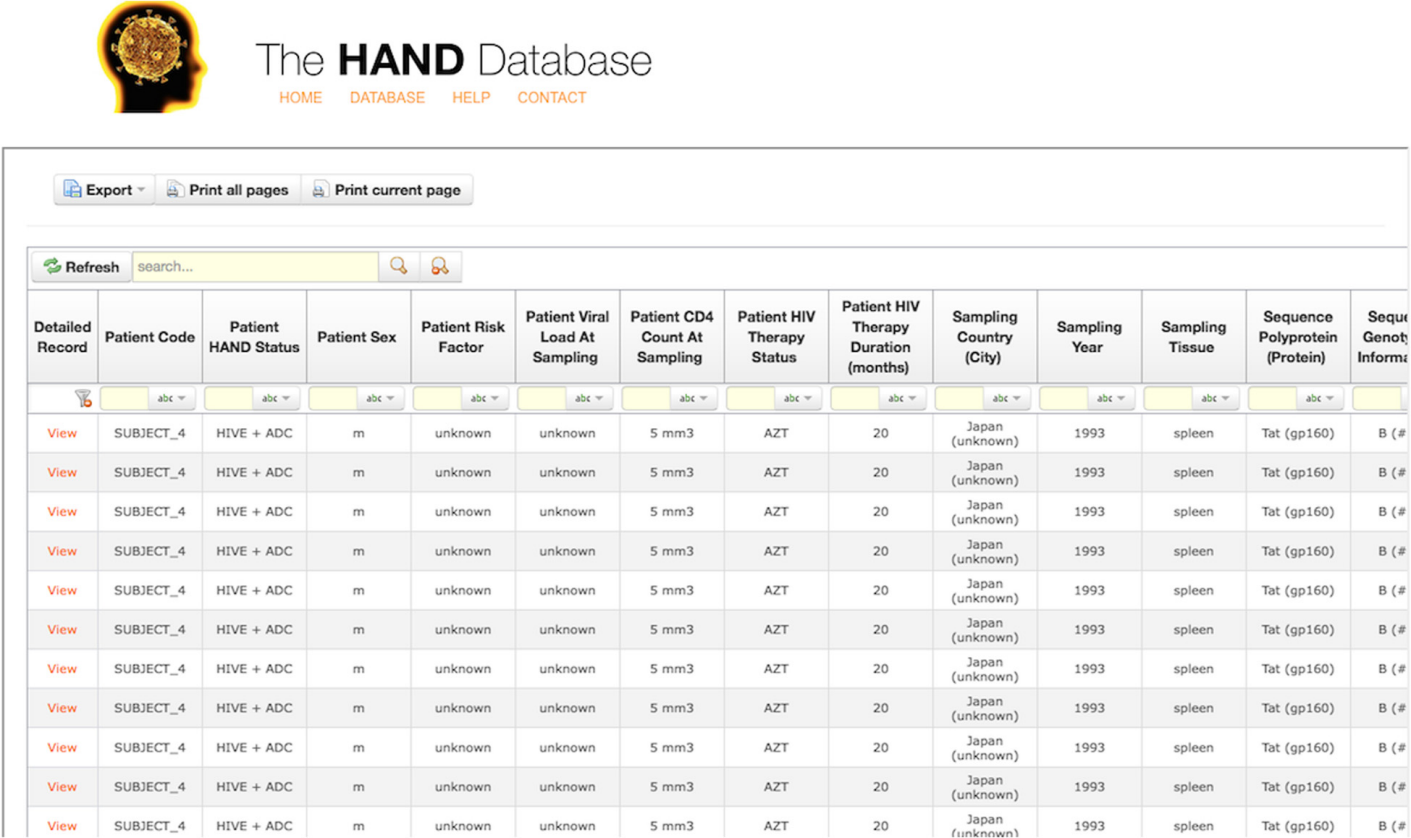
The HAND Database Search Interface. The HAND Database provides flexible searching, filtering, and browsing capabilities. Sequence entries and annotations of interest can be exported into a variety of file formats for further use. In addition, a website navigation bar allows easy access to help, contact, and background information pages

The HAND Database was developed into a publically available, web accessible resource. The database website provides a home page with background information on HAND, as well as a help page to assist with database navigation (Fig. 1). The database itself allows for easy querying and downloading of user-defined data subsets. Researchers can perform a simple search using a keyword, or employ multiple column filters for a custom-made data subset. Selected entries can subsequently be downloaded into a variety of formats at the user’s discretion. Additional features include sorting by annotation of interest, as well as an option for viewing the complete record for any given entry.

### Database content

**Fig. 2:**
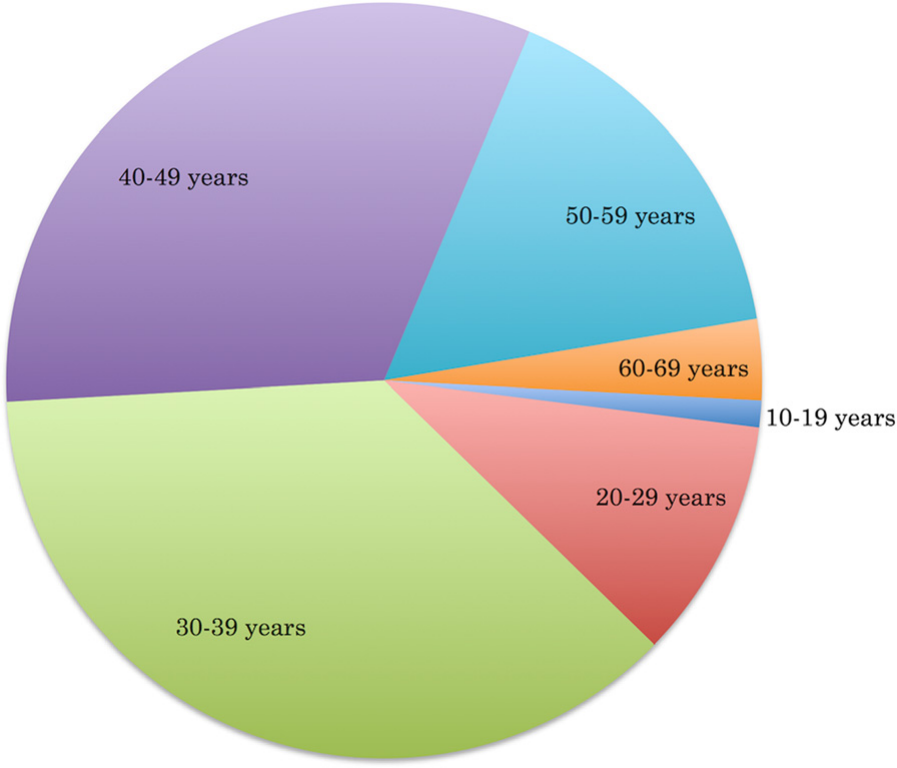
Distribution of HAND Database Entries By Age. Age distribution across database individuals showed 69 % of individuals for whom this information was available were between the ages of 30 to 49 years

**Fig. 3:**
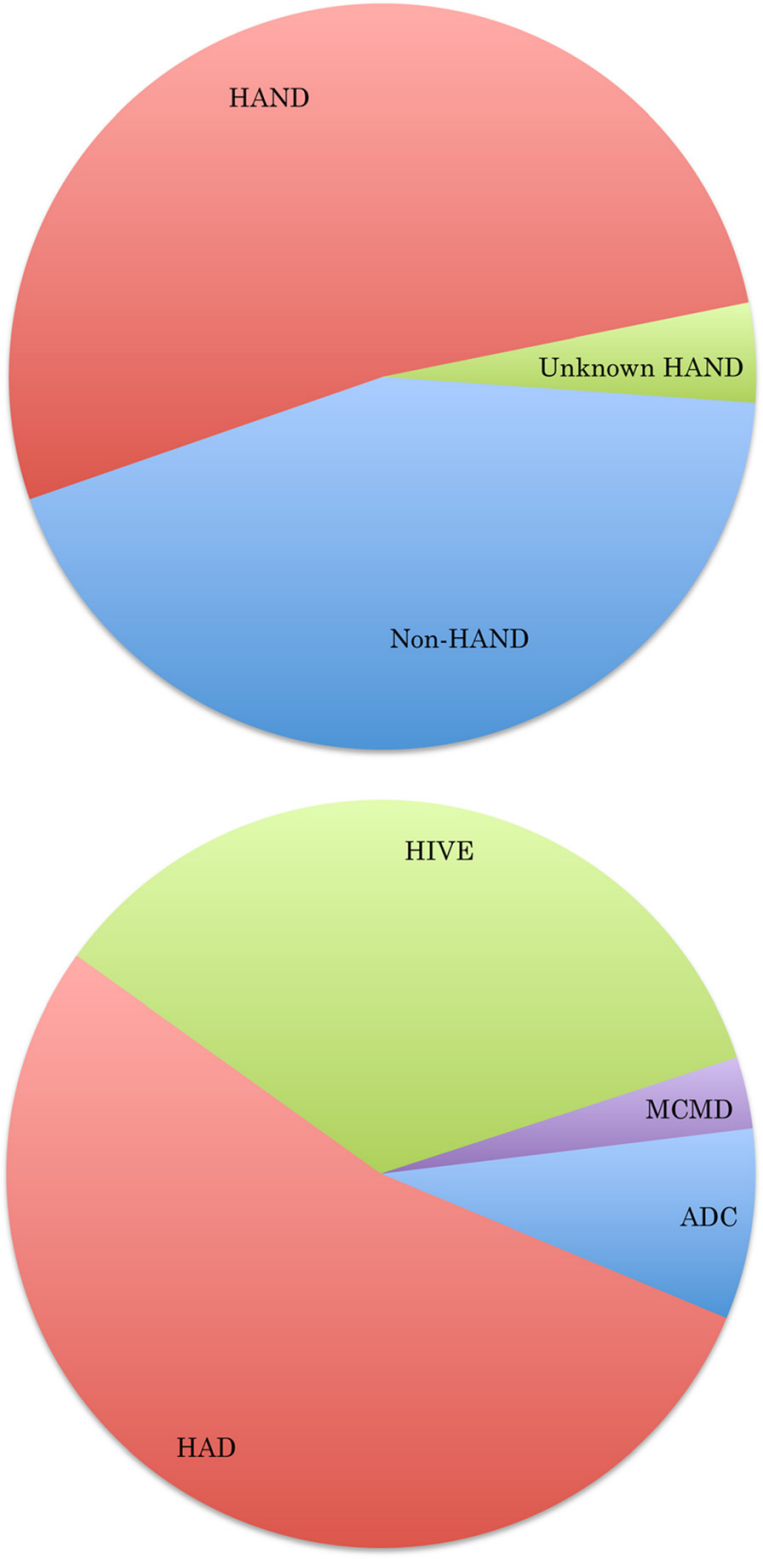
Distribution of HAND Database Entries By HAND Status And HAND Type. The top chart shows HAND status distribution across all database individuals, and the bottom chart shows HAND type distribution across database individuals for whom this information was available. The majority of individuals with HAND had HIV-associated dementia (HAD), followed by HIV-encephalitis (HIVE), AIDS dementia complex (ADC), and minor cognitive-motor disorder (MCMD). HAND type designations were obtained from the literature, and for some individuals, more than one HAND type had been assigned

**Fig. 4:**
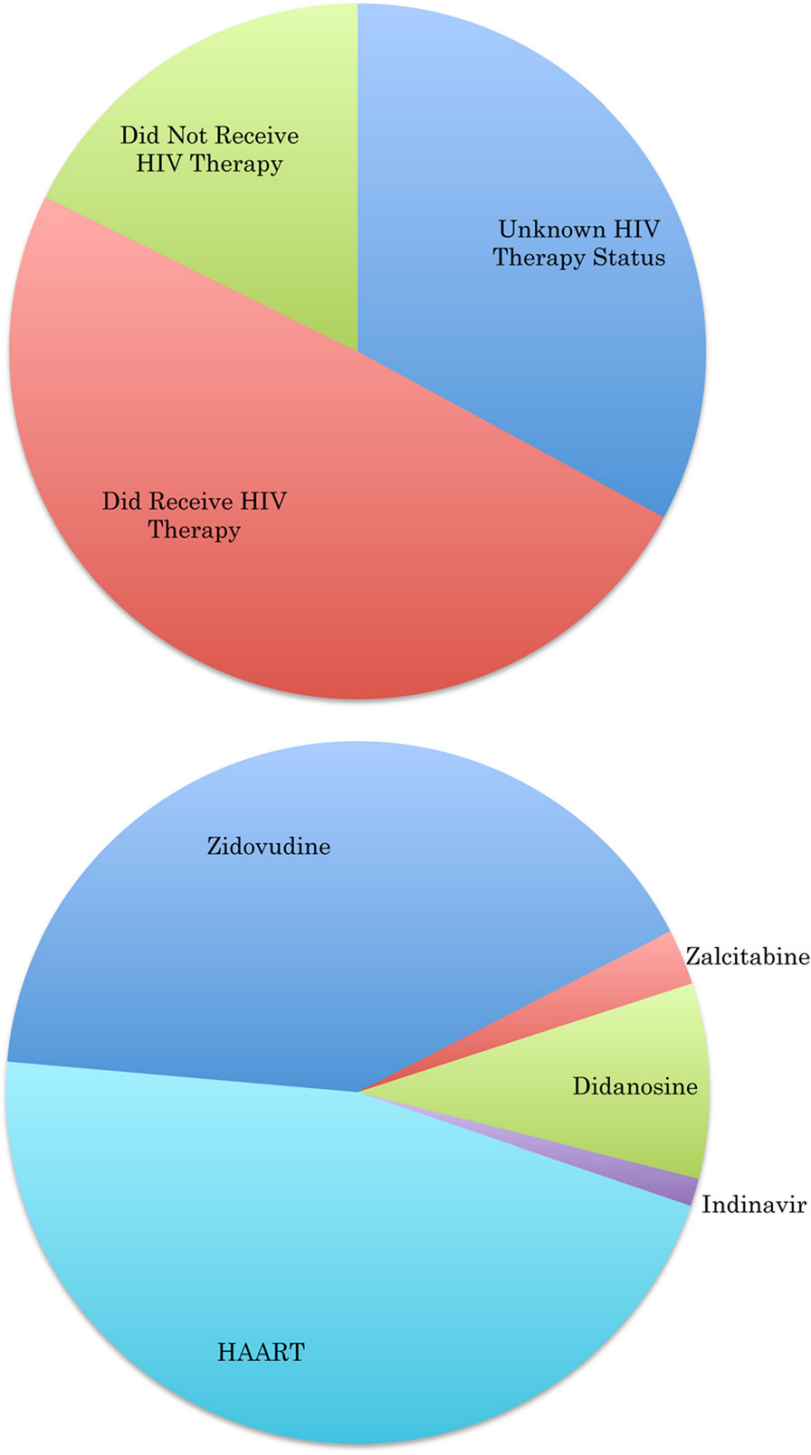
Distribution of HAND Database Entries By HIV Therapy Status And HIV Therapy Type. The top chart shows HIV therapy status distribution across all database individuals, and the bottom chart shows HIV therapy type distribution across database individuals for whom this information was available. Nearly half of all treated individuals had received HAART. Therapy type designations were as we found to be reported in the literature, and for some individuals, more than one HIV therapy type had been assigned

The HAND Database currently contains 5,783 HIV-1 sequences, representing a total of 163 unique individuals assessed for HAND status. For the 87 individuals with age information available, ages ranged from 19 to 63 years, with the largest proportion of individuals between 30 and 49 years of age (69 %) (Fig. 2). Gender information was available for 64 individuals, the majority of whom were males (77 %). HAND status, the absence or presence of HAND, was obtained for almost all database individuals (96 %), and indicated a close split between non-HAND (44 %) and HAND (52 %) patients. The top three reported HAND types in HAND-positive individuals were HIV-1-associated dementia (HAD, 54 %), HIV-1 encephalopathy (HIVE, 35 %), and AIDS dementia complex (ADC, 8 %) (Fig. 3). HIV treatment status information, whether or not an individual had received HIV treatment prior to sampling, was available for 67 % of individuals, and the majority of individuals with treatment information had received some form of treatment (49 %). Nearly half of all treated individuals had received HAART (46 %) prior to sampling, while the rest had received one or more forms of HIV monotherapy (54 %) (Fig. 4).

**Fig. 5:**
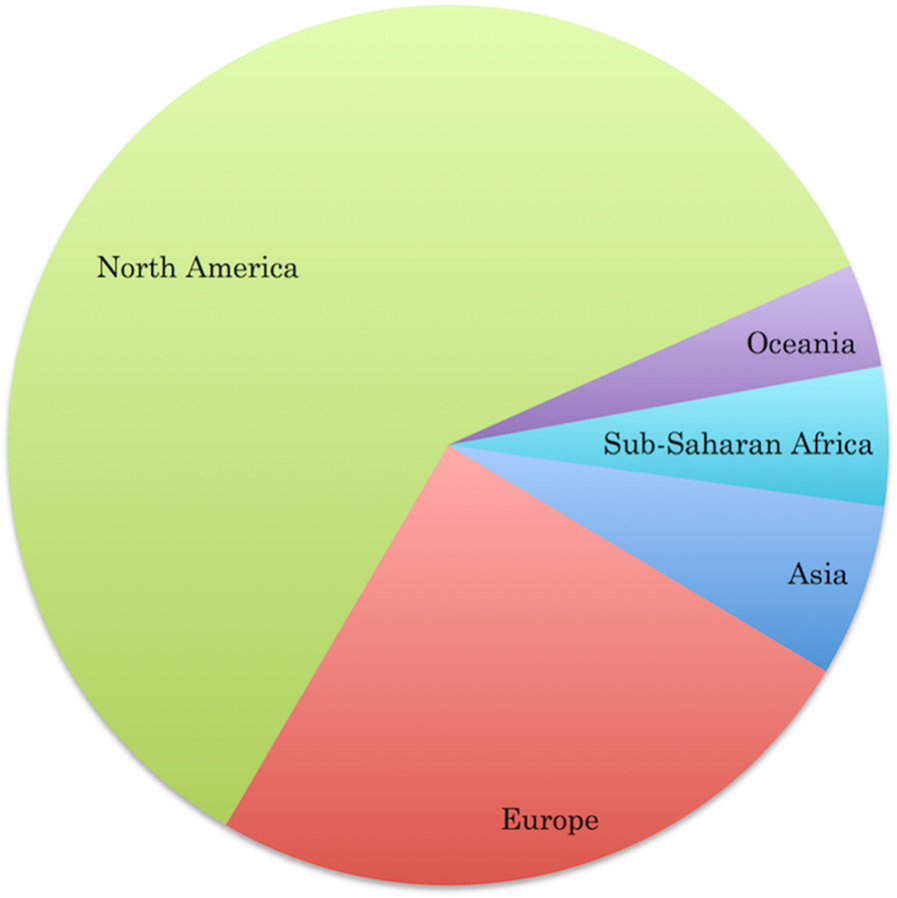
Distribution of HAND Database Entries By Sampling Geographical Region. Sampling geographical region distribution across database individuals showed the majority of database sequence entries for whom this information was available were derived from North American samples

Geographical region sampling information was available for 156 patients, with the top three sampling regions being North America (60 %), Europe (25 %), and Asia (6.4 %) (Fig. 5). Samples were derived from 20 different tissue types, with the top three sampling tissues being brain (47 %), lymph node (14 %), and CSF (7 %).

**Fig. 6:**
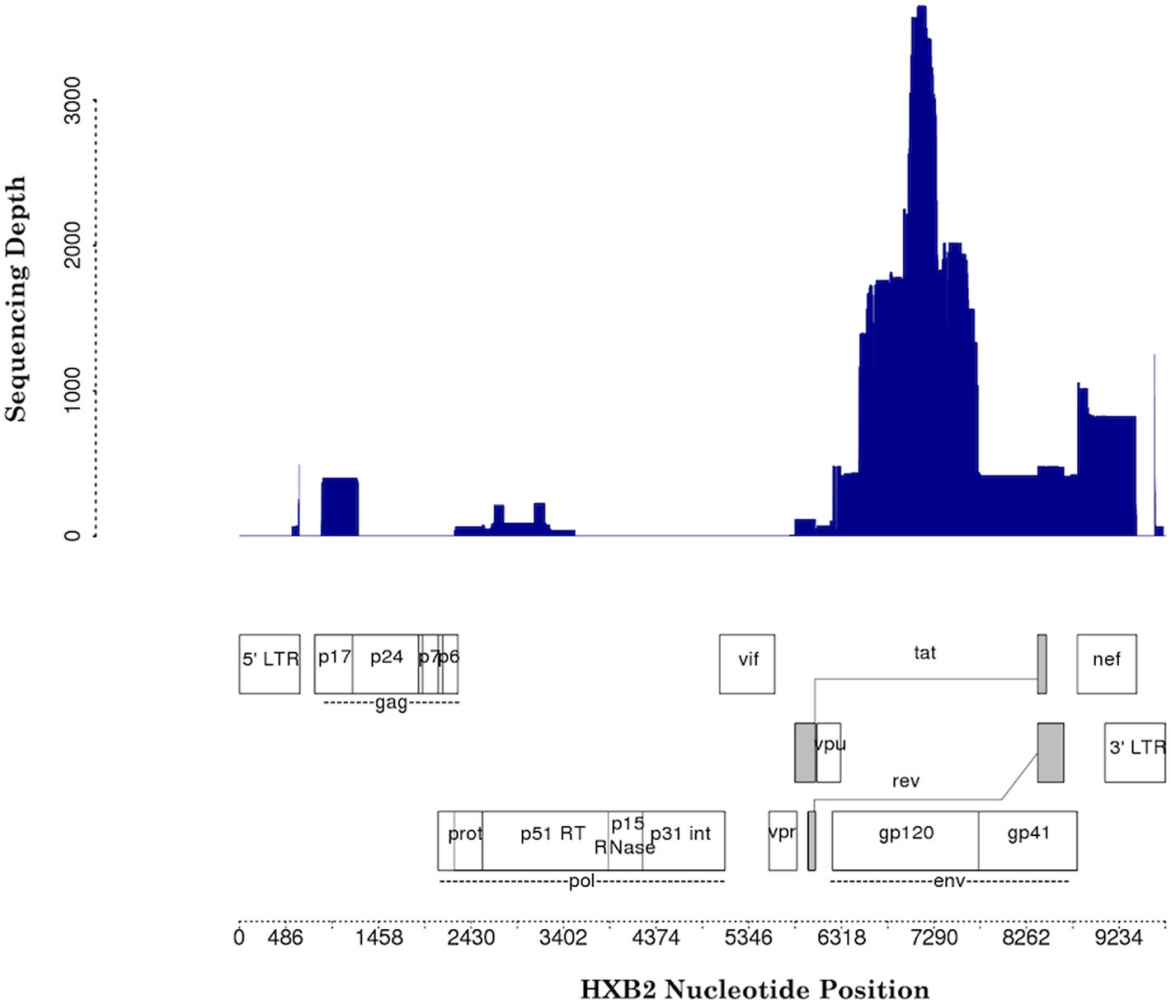
HAND Database HIV-1 Genome Coverage And Sequencing Depth. The top panel displays HAND Database sequencing depth across the HXB2 reference sequence, and the bottom panel displays HIV-1 gene location across the HXB2 reference sequence. The *env* gene was the HIV genomic region with the greatest sequencing depth. HXB2 accession number: K03455

Five HIV-1 genes were represented in our database, *gag*, *pol*, *env*, *tat*, and *nef*, with the majority of sequence coverage in the *env* gene (Fig. 6). This result was expected due the known role of *env* in macrophage tropism, viral replication, and activation of pro-inflammatory responses toward neuronal injury [19–21]. Of all archived sequences, 79 % of sequences that underwent genotyping validation were of the pure B subtype, and all non-recombinant sequences were confirmed as having been correctly reported in the literature. Sixteen sequences were found to have undergone recombination events not reported in either the source literature or databases.

## Discussion

Despite increased HAND research and treatment efforts, the persistent prevalence of HAND continues to pose a great challenge to the HIV research and patient communities. Investigation in this area is limited by small sample sizes, primarily due to difficulty in obtaining tissue samples, and by variation in study protocols and result interpretation. Furthermore, errors and inconsistency in HIV genotyping compound the complexity in delineating viral mechanisms toward neuropathology. The HAND Database described here serves to narrow these research gaps and addresses the need for a reliable and centralized HAND data source for advanced research purposes.

The HAND database contains up-to-date and well-curated HAND virus and patient information. All sequence data have been subject to stringent quality control examination and re-genotyping, thereby laying a solid foundation toward elucidation of viral mechanisms driving neuropathology under various epidemiology settings.

In creating this resource we noted a number of sequencing and sampling biases that currently limit research in the area, and have developed a set of potential research directions that may greatly benefit the HAND research community. First, although prior studies have indicated the role of multiple HIV proteins, including Nef, Vpr, and Tat [22–29], toward HAND development, the majority of research in the area has focused on the gp120 envelope glycoprotein. This sequencing bias is largely due to interest in Env for its role in conferring viral tropism for microglia and macrophage cells [30–33], its role in non-neuronal cell replication [34], and for its potential as an HIV therapeutic target [35]. A shortage of sequence data beyond the *env* gene, however, limits our ability to perform data-driven HAND research on the complete viral genome, and therefore an increase in sequencing efforts in other areas of the genome would provide insight into the role of regulatory and accessory proteins toward HAND pathogenesis. Second, there is a distinct lack of sequence data from HIV epidemiologically important regions including many Asian and Sub-Saharan African countries (Fig. 5). Limited access to HAART contributes to an increased vulnerability of HIV individuals in these geographical regions to the most severe forms of HAND. Recent studies indicate HIV-associated dementia (HAD) affects over 25 % of HIV individuals in several Sub-Saharan African countries [36–38]. In addition, research on treatment-naïve HIV-1-individuals in Thailand has greatly contributed to our understanding of HAND pathogenesis [39]. Finally, we noted a bias toward sequencing of male individuals. Research beyond the HIV field has implicated gender as playing a role in determining those genetic processes leading to neurocognitive deficiencies [40, 41]. A lack of information on HAND females, however, currently proves an obstacle in determining potential gender differences in HAND pathogenesis.

## Conclusions

Developing a better understanding of mechanisms underlying the development of neurocognitive disorders is crucial toward providing the HIV patient community with a higher quality of life, and toward prevention of enhanced transmission. Through consolidation and validation of data from multiple data sources, here we have developed the HAND Database, a single, intuitive platform from which researchers can launch their high-throughput HAND sequencing projects. The HAND database contains up-to-date and curated HAND HIV virus and HIV-infected individual information, providing a solid foundation toward the elucidation of viral mechanisms driving this neuropathology. In particular, we anticipate this database will be of great use in increasing HAND research efforts in resource-limited countries. We plan to continue expanding the HAND Database as new HAND viral sequence data become publically available.

## Availability and requirements

All records are freely available and accessible at www.handdatabase.org.
